# A Case of Drowning

**Published:** 1894-02

**Authors:** T. C. Osborn

**Affiliations:** Cleburne, Texas


### TEXAS MEDICAL JOURNAL.

ESTABLISHED JULY, 1885.

PUBLISHED MONTHLY.—SUBSCRIPTION $2.00 A YEAR.

VOL. IX. AUSTIN, FEBRUARY, 1894. NO. 8.

### Original Contributions.

For Texas Medical Journal.

### A CASE OF DROWNING.

BY T. C. OSBORN, M. D., CLEBURNE, TEXAS.

Prepared to be read before the Johnson County Medical Society in December, 1893.

THE rarity of resuscitation from the asphyxiated result of drowning, makes an interesting subject for discussion by medical men; and as there is so much that is unsettled involved in it, any reports of cases resuscitated after submersion, it seems to me, ought to be registered in the statistics of the age. I will, therefore, offer no apologies to the society for requesting the insertion of this report in the transactions, nor make any claim of originality in the methods adopted to establish reanimation.

Strangely enough, as in this case, statistics will sustain my assertion that one per cent. of the total number of cases reported, little children, are the victims of the wash tub, water barrel or neighboring tank at home.

My case happened here in Cleburne in the autumn of the year 1890, and was in the person of a baby boy about two years of age, the child of Mr. C. L. Mackelhany, who is now a citizen of Erath county, but who, at the time of the accident, was my next door neighbor, not over sixty feet from my residence, and, of course, in easy reach of my assistance. The child was playing alone in the yard, near a wash tub filled with stock water, and in some way tumbled head foremost into it. How long he had been there is uncertain, as his mother, in passing, saw him buried head downwards in the water, with no movement in any part of the body. In this condition she seized him by the feet, which she placed under her left arm, letting his head hang down, and ran through the house to the front door, where she stood frantically screaming at top of her voice, alarming the entire neighborhood. She had been standing there full two minutes before I reached the door. Inferring at a glance the nature of the trouble, and thankful for the help she was unconsciously giving by holding the child in the proper position for the water in the stomach and lungs to run out, carried it hastily to a bed-room, stripping the clothes off, as I went along, and calling for towels and hot flannels to other members of the family, who were apparently as deaf as the mother, and chorusing her screams in terrible discordance. These screams, however, had the effect of bringing into the house quite a number of intelligent ladies who immediately gave me all the assistance needed, and very soon the towels and flannels were in use, and some were rubbing the upper and lower extremities with dry mustard to stimulate, if possible, a circulation in the surface, whilst I, on my part, was at work raising and lowering the arms to induce respiration; and finding no response, I attempted to insufflate the lungs by applying my mouth to his, but found it impossible to do so, on account of what I knew must be retraction of the tongue over the elevated glottis. I thrust my finger well back in the fauces, and pressed the stiffened tongue downwards and forwards from off the air passage. When this was performed there was a perceptible flow of air into the lungs, and I withdrew my finger to raise and lower the arms again, thinking there would be no further obstruction to the entrance of the air. In all this time there was no sign of life. The body was cold in all its parts; the skin had everywhere a death-like pallor, and in many places sodden, and with cutis anserina strongly depicted; but there was no cadaveric rigidity except in the cold tongue and cold eyeballs, turned backward and set, whilst the lids were closely drawn together, and could only be separated by force. There was total insensibility in all parts of the body; nor was there a pulse to be found, nor the sound of a heart beat, when my ear was applied, over the cardiac region. The body was apparently dead, and had been so for no telling how long a time.

In the second year of my professional career, 1841, a pig, feeding near me, fell dead which, for a few minutes, I attempted to bring back to life, but with no success. The suddenness of the death excited my curiosity to know the cause of it. For this purpose I made a post mortem, and on opening the thorax I was astonished to find the heart still throbbing. I removed that organ from the body—there was no bleeding—and placed it in a pan of warm water, where it continued to pulsate for the next ten minutes, the motions growing fainter and fainter, until it ceased entirely. And the memory of that event led me to hope that the child's heart might still be beating, but so feebly as to make no sound. With this hope I again pressed the tongue out of the way, and again there was a perceptible rush of air into the lungs in almost a sob. At this point I was sanguine in my hopes, and again released the tongue to assist expiration; but the life did not seem to return according to my expectations. Then for the third time I pressed the tongue and held it there, as I should have done at first. It was now less resisting, and upon opening the passage there was a gasp, the eyelids partially unclosed, the balls seemed trying to move, and to my great satisfaction respiration began; the eyes opened, the balls righted up, and, after the second or third act of breathing, a gasp followed, and the child uttered a vigorous scream. As soon afterwards as possible I gave a teaspoonful of strong brandy, and some of it getting into the glottis, caused convulsive strangling in which, apparently, every muscle in the body was actively at work. Ah, this was the supreme moment of my elation. I knew now the circulation would be aroused to renewed life, and I felt amply compensated for my persistent efforts in the restoration of life.

Such instances make much the largest compensation in the feelings of the doctor. Life was gone; life was restored, and that by efforts unmistakably his own. It is a glorious feeling. Of course it is understood that not as much time was taken up in the efforts at resuscitation as I have occupied in detailing it. In fact I feel quite sure that not more than five minutes, at the outside, was passed through in the work. But, after all, I must confess my conviction that the time would have been materially shortened if I had held down the tongue with the first pressure instead of releasing it to use my hands in effecting expiration.

In bold relief to the picture here presented was the gentle mother, standing statue-like where I had met her, at the front door, with unwinking eyelids and dilated pupils, looking straight ahead, as if viewing something over the border, far into the nineteenth century, and with every breath uttering a high-pitched scream, perfectly indifferent to passing friends whose efforts at consoling her were as totally wasted as if her ears had lost all sense of hearing. Nor did she cease, or change position until I went out and shook her arm, asking if she had gone crazy? Then, for the first time, she realized her condition, turned silently into the hall, took the child in her arms, and smothered him with kisses and embracements. My parting instructions to her were, "make him drunk and let him sleep it off, and no harm will happen after he awakens."

There is, in my opinion, but little doubt that asphyxia is the cause of death in 90 per cent. of the cases of drowning by accident, as the post mortem appearances in nine cases of ten show the presence of excess of water in the stomach by the act of swallowing; with frothy mucus in the bronchial passages, and infiltration of water in the air tubes and cells of the lung tissue.

It would seem an easy inference that the lungs would be more or less collapsed, owing to the last expiration having expelled all the air enclosed; but this is not always true. On the contrary, in a number of cases the lungs are found distended to their utmost capacity, being filled with water, and although some air remains, the greater bulk of the substance is filled with water, which causes them to have increased dimensions. Some observers have ascribed death in drowning to a congested state of the vessels of the brain, and that death takes place in most cases by a species of apoplexy; but it is my belief that the obstruction to the passage of the blood through the lungs is quite sufficient to explain the reason why we see congestion in the vessels of the brain in bodies that are drowned; and the congestion is probably an after effect to the interruption of the cerebral functions.

The most characteristic appearance of death from apoplexy is extravasation of blood on the brain, a condition which is rarely seen in the drowned; and probably when it does exist, it may be traced to mechanical violence before submersion, or to the head colliding with hard bodies in falling into the water.

One of the most constant appearances found in post mortems after drowning, is the distended condition of the cavities of the heart with dark blood, which is a positive evidence of death from asphyxia.

The period at which death takes place by drowning is an interesting question, and as it sometimes becomes a medico-legal investigation, has to be answered by eliminating all cases of death by syncope, or apoplexy; for in these the circulation and respiration are simultaneously arrested.

How long can a person remain under water without becoming asphyxiated? And at what length of time under submersion may we hope for resuscitation?

The answer to the first depends mainly upon the length of time the person may be able to hold his breath, and this is so different with different persons that the question must necessarily remain undetermined. But it may be safely affirmed that no one can cease breathing for longer than two minutes without becoming asphyxiated. The heart, however, may continue to beat in a feeble manner for several minutes after all other signs of death have become apparent, an instance of which is given above of the pig. This fact made the basis of my hope with the little Mackelhany.

Many cases, on the other hand, are reported where death occurred in one minute and a half after being submerged; and in others, as long as ten minutes, or even fifteen minutes after drowning. The woman who was exhibited in London in 1882, who could hold her breath under water for two and a half minutes, is certainly a remarkable exception to the general rule.

The reply to the second question is of practical importance to physicians in their efforts at resuscitation. The insensibility which follows the submersion gives to the body the likeness of death, although under the water but a short time; but this apparent death should not deter us from making every possible effort to restore animation. The longest instance recorded with any claim to authenticity, is one in which a woman is stated to have recovered by prompt treatment after a submersion of twenty minutes. It is, however, my own conviction that no one has ever recovered after the heart has entirely ceased its pulsations.

The treatment of drowned bodies requires first of all a cool and steady head on the part of the attendant. He must not lose his head for a single instant, and he should have at his fingers ends, as it were, all the methods and facilities to cover the case, and his activity and perseverance must do the rest.

Marshall Hall is good authority the world over, and he lays down the following rules, which I will quote without apology, for the reason that we cannot too well know them when the demand is made upon our skill:

1st. The wet clothing must be removed.

2nd. Wipe the surface dry and cover it with warm flannels.

3rd. Clear the mouth, nostrils and throat of all mucous froth, or of other substances likely to interfere with respiration; pull forward the tongue *and keep it there*, so that it may not fall back and cover the wind pipe. (This, in my opinion, is the most important part of the management.)

4th. Place the body at full length with the face downward, the forehead resting on one arm.

5th. Apply ammonia, aromatic vinegar, snuff, or other stimulants to the nostrils. (This is a uselss step, at least until respiration is partially regained.)

6th. If respiration is not quickly restored spontaneously then the body should be placed upon its back with the head slightly raised. The arms should be carried outward and upward from the chest, raised above the head and held that way for two seconds to induce inspiration; then lowered and brought closely to the sides of the chest so that expiration may be effected. Pressure on the lower end of the sternum aids in the expiratory action. This movement should occupy two seconds. These alternate movements should be persevered in for twelve to fourteen minutes, or until respiration is re-established. When the power of swallowing returns, warm water and brandy may be given, and the patient allowed to sleep. This treatment may be continued for some hours, except in those cases in which the body has been under the water a long time, and is taken out cold and stiff.

I would be pleased if, in the discussion on this case, you would impress me with the length of time my patient was asphyxiated, and with the experience others of the society have had with patients drowned.

